# The interactome and spatial redistribution feature of Ca^2+^ receptor protein calmodulin reveals a novel role in invadopodia-mediated invasion

**DOI:** 10.1038/s41419-017-0253-7

**Published:** 2018-02-20

**Authors:** Tao Li, Li Yi, Long Hai, Haiwen Ma, Zhennan Tao, Chen Zhang, Iruni Roshanie Abeysekera, Kai Zhao, Yihan Yang, Wei Wang, Bo Liu, Shengping Yu, Luqing Tong, Peidong Liu, Meng Zhu, Bingcheng Ren, Yu Lin, Kai Zhang, Cheng Cheng, Yubao Huang, Xuejun Yang

**Affiliations:** 1 Department of Neurosurgery, Tianjin Medical University General Hospital, Tianjin, 300052 China; 2 Laboratory of Neuro-Oncology, Tianjin Neurological Institute, Tianjin, 300052 China; 3 Key laboratory of Post-trauma Neuro-Repair and Regeneration in Central Nervous System, Ministry of education, Tianjin, 300052 China; 4 Tianjin key Laboratory of Injuries, Variations of Regeneration of Nervous System, Tianjin, 300052 China; 5 The university of Texas MD Anderson cancer center, Neuro-Oncology Department, Houston, TX 77030 USA; 6 Department of Physiology and Pathophysiology, Tianjin Medical University, Tianjin, 300070 China; 7 Department of Neurosurgery, The Affiliated Hospital of Logistics University of PAP, Tianjin, 100039 China; 8 Department of Neurosurgery, The Affliated Hospital of Qingdao University, Qingdao, 266003 China

## Abstract

Numerous studies have shown that calmodulin (CaM) is a major regulator of calcium-dependent signaling, which regulates cell proliferation, programmed cell death, and autophagy in cancer. However, limited information is available on mechanisms underlying the effect of CaM on the invasive property of glioblastoma multiforme (GBM) cells, especially with respect to invadopodia formation. In this study, we find that CaM serves as a prognostic factor for GBM, and it is strongly associated with the invasive nature of this tumor. Results of preliminary experiments indicated that CaM concentration was significantly correlated with the invasive capacity of and invadopodia formation by different GBM cell lines. CaM inhibition via a small hairpin RNA or a pharmacological inhibitor significantly disrupted invadopodia formation and MMP activity and downregulated vimentin expression. Moreover, CaM knockdown exerted a strong anti-invasive effect on GBM in vivo. Interestingly, epidermal growth factor treatment promoted CaM redistribution from the nucleus to the cytoplasm, eventually activating invadopodia-associated proteins by binding to them via their cytosolic-binding sites. Moreover, CaM inhibition suppressed the activation of invadopodia-associated proteins. Thus, our findings provide a novel therapeutic strategy to impede GBM invasion by inhibiting invadopodia formation, and shed light on the spatial organization of CaM signals during GBM invasion.

## Introduction

Glioblastoma multiforme (GBM) is the most common and one of the most malignant primary brain tumors occurring in humans. The major obstacle in GBM treatment is diffuse tumor invasion, which allows glioma cells to escape complete surgical resection and chemotherapy and radiation therapy^1,2^. Therefore, it is imperative to identify effective therapeutic targets that can impede GBM invasion to improve the poor prognosis of GBM.

Degradation of extracellular matrix (ECM) promotes tumor invasion, and invadopodia are critical for ECM degradation. Invadopodia are electron-dense, actin-based dynamic protrusions of the plasma membrane of metazoan cells, including invasive cancer cells, that induce ECM degradation^3^. Invadopodia allow cancer cells to couple ECM degradation with motility, thus facilitating their migration through the tissue microenvironment. Moreover, invadopodia formation is correlated with the ability of cancer cells to invade and metastasize^3^. Thus, invadopodia formation is a critical hallmark of tumor cells that undergo systemic dissemination and metastasis^4^. Accumulating evidences indicate that abrogation of invadopodia formation in human cancer cells greatly limits their migratory and/or invasive abilities^5,6^, suggesting that targeting invadopodia formation is a promising strategy to prevent cancer cell invasion.

Calmodulin (CaM), a calcium (Ca^2+^)-trigger protein with four EF hands, is highly conserved and regulates several enzymes, ion channels, aquaporins, and other proteins through Ca^2+^. CaM–Ca^2+^ complex stimulates several protein kinases and phosphatases, some of which are associated with cell migration and invasion^7^. Moreover, in response to various signals, the rapid redistribution of CaM caused by interaction with p68 RNA helicase contributes to a series of cellular processes, including cell motility^8–10^. Although multiple studies have confirmed the important role of CaM in linking Ca^2+^ signaling with cell motility^11,12^, limited information is available on the relationship between CaM and invadopodia formation and on the effect of CaM redistribution on GBM cell invasion in response to extracellular signals.

In this study, we found that CaM promoted GBM cell invasion by potentiating invadopodia formation. CaM inhibition by using a pharmacological inhibitor or by silencing of the CaM gene effectively abolished GBM invasion and invadopodia assembly. Moreover, we unexpectedly found that extracellular signals such as epidermal growth factor (EGF) facilitated CaM translocation from the nucleus to the cytoplasm and contributed to the cytosolic activation of invadopodia-associated proteins. These results indicate that CaM can serve as a therapeutic target to impede cancer cell invasion by inhibiting invadopodia formation, and provide information on the spatial organization of CaM signals during GBM invasion.

## Results

### CaM expression in glioma tissue specimens and glioma cell lines

**Fig. 1:**
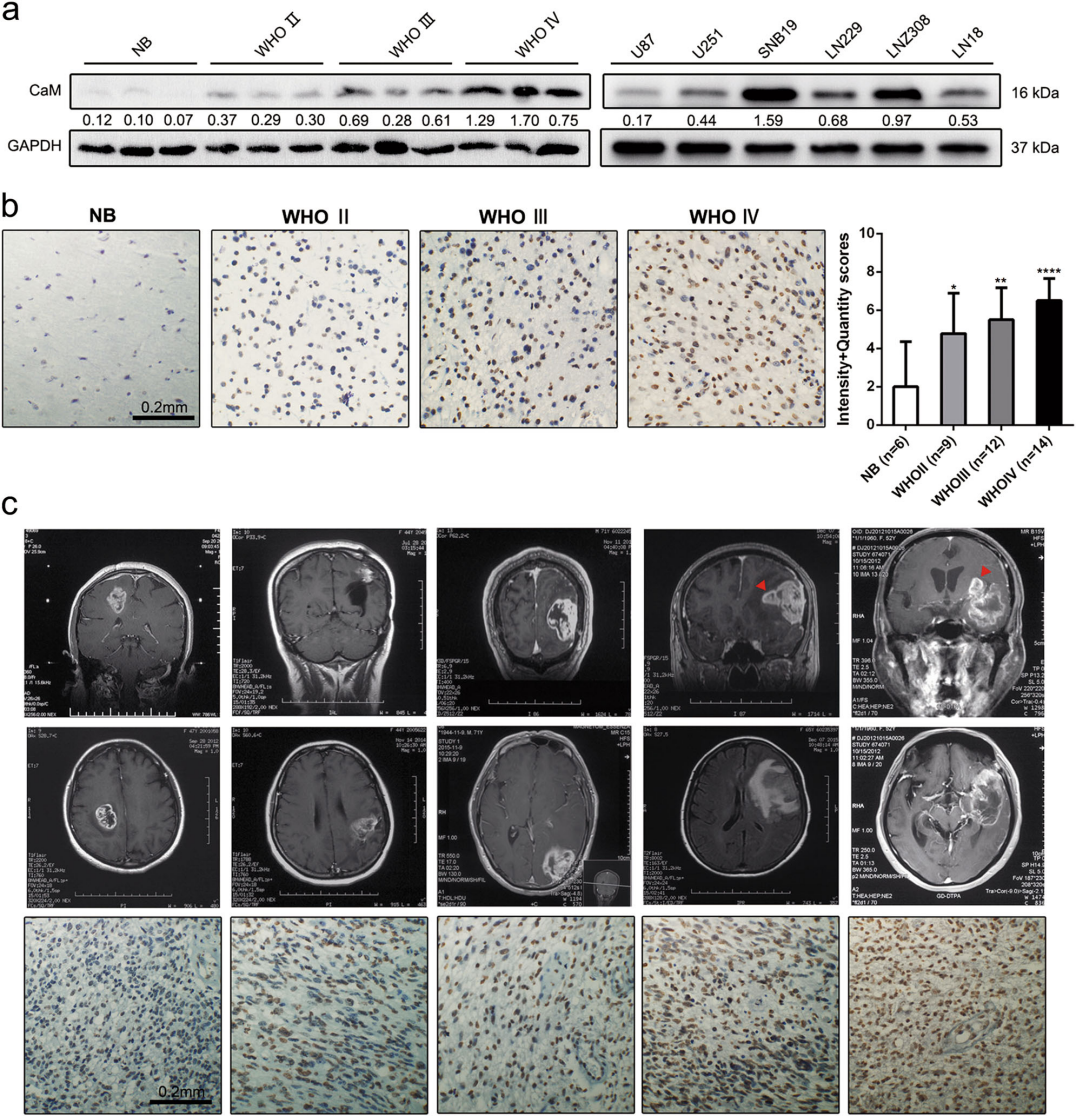
The expression of CaM in glioma specimens and glioma cell lines. **a** CaM protein levels were higher in GBM (*n* = 3) than in lower-grade glioma (WHO Grade II: *n* = 3, WHO Grade III: *n* = 3) and normal tissues (*n* = 3), as determined by western blot. **b** CaM expression was increased in GBM samples (*n* = 14) compared to lower-grade glioma (WHO Grade II: *n* = 9, WHO Grade III: *n* = 12) and normal brain tissues (*n* = 6) using immunohistochemical staining (Data are presented as means ± SDs of three different microscopic visions from every independent experiment, *p < 0.05, **p < 0.01, ****p < 0.0001). **c** Five GBM patients’ MRI images with representative images of immunohistochemical staining of CaM (Images were taken from three different microscopic visions from every independent experiment, Red triangle: disseminated tumor)

Multiple studies indicate that the level of expression of CaM is elevated in tumor cells compared with that in cells derived from normal tissues^13–16^. To determine CaM expression in gliomas, we first performed western blotting analysis using glioma cell lines U87-MG, U251-MG, LN229, SNB19, LN308, and LN18; glioma tissue specimens; and normal tissue specimens. Clinical specimens included in this study are listed in Supplementary Table 1. CaM expression was elevated in GBM tissue specimens (*n* = 3) compared with that in normal tissue (*n* = 3) and lower-grade glioma (LGG) (WHO grade II, *n* = 3, and WHO grade III, *n* = 3) specimens (Fig. 1a). Immunohistochemistry (IHC) analysis was performed to assess the level of expression of CaM in glioma tissue specimens (WHO grade II, *n* = 9; WHO grade III, *n* = 12; and WHO grade IV: *n* = 14) and normal tissue specimens (*n* = 6). Representative immunohistochemical staining patterns for CaM were illustrated, with GBM tissue specimens showing high CaM expression. based on this result, we calculated the immunoreactive scores of the different tissue specimens (Fig. 1b). To further confirm the clinical relevance of CaM expression, we detected *CALM1* and *CALM2* expression in the clinical specimens using two antibodies (HPA044999 and CAB018558) from the human protein atlas (www.proteinatlas.org). We detected negative or weak CaM expression in glial cells obtained from normal tissue and LGG specimens and median positive or strong CaM expression in GBM cells (Supplementary Figure 2a–b).

Immunostaining analysis was performed using five GBM specimens showing varying stages of the disease and different degrees of invasion. MR images are shown in Fig. 1c. Results of IHC analysis detected CaM overexpression in GBM specimens showing diffuse invasion. These results suggest that CaM is a prognostic factor for GBM and plays a vital role in the aberrantly extensive spread of GBM.

### CaM is associated with the poor prognosis and aggressive nature of GBM

**Fig. 2:**
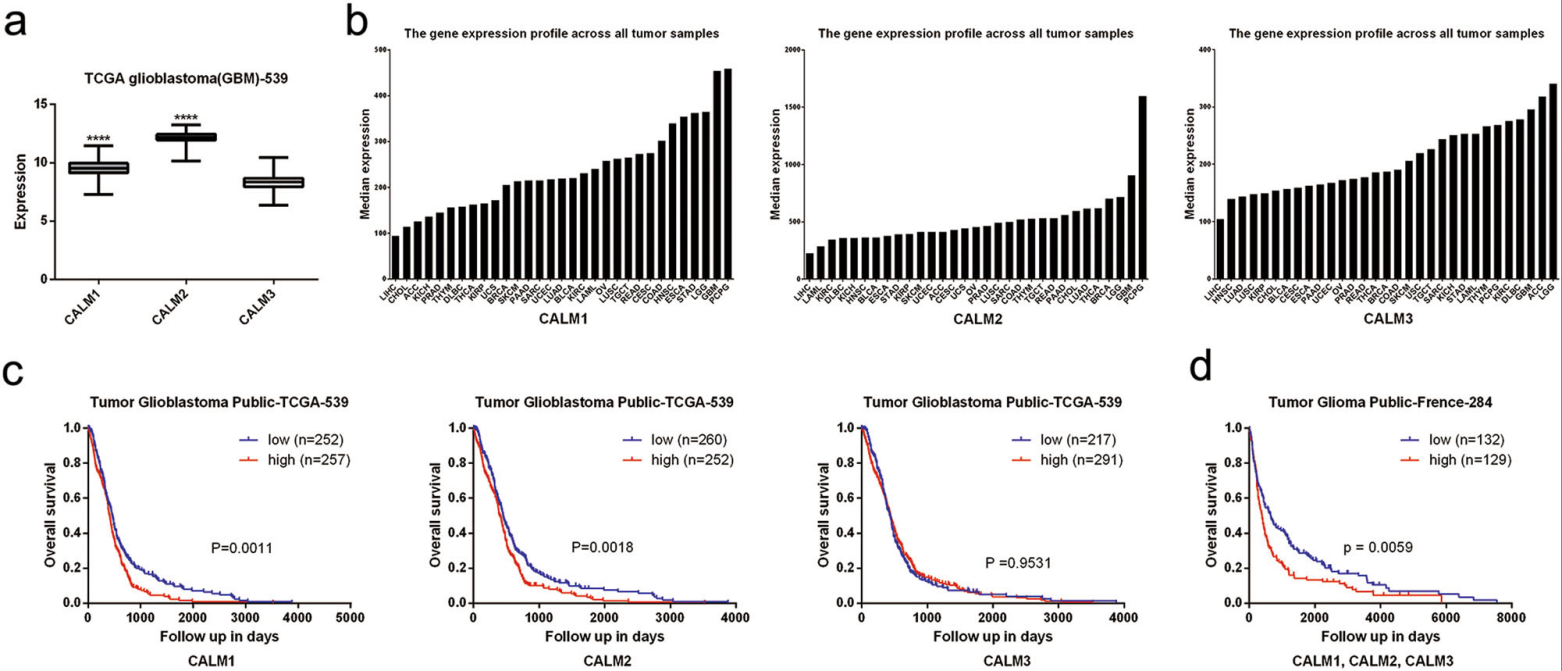
CaM was associated with poor prognosis and aggressive nature in GBM. **a** *CALM1*, *CALM2* and *CALM3* mRNA expression in the TCGA GBM data set (*CALM1*: *n* = 523,* CALM2*: *n* = 523, *CALM3*: *n* = 523, ****p < 0.0001). **b** *CALM1*, *CALM2* and *CALM3* expression profile across TCGA pan cancer data set. Images were taken from the GEPIA (Gene Expression Profiling Interactive Analysis) online database (http://gepia.cancer-pku.cn). **c** Kaplan–Meier representation of the overall survival of the two groups of patients with high or low *CALM1*, *CALM2* and *CALM3* expression according to TCGA GBM data set. Statistical analysis was performed with the log-rank test. **d** Kaplan–Meier representation of the overall survival of the two groups of patients with high or low CaM genes expression according to French glioma data set. Statistical analysis was performed with the log-rank test

Three human CaM genes, namely, *CALM1*, *CALM2*, and *CALM3*, encode CaM proteins with an identical amino acid sequence, and each CaM gene shows tissue-specific relative transcriptional activity^17^. To select small hairpin RNAs (shRNAs) that effectively suppressed CaM expression in GBM cells, we analyzed a TCGA GBM microarray data set of 539 patients. Compared with the mRNA level of *CALM3*, mRNA level of *CALM1* and *CALM2* was significantly increased in GBM cells (Fig. 2a).

We further assessed CaM expression in a TCGA pan-cancer data set obtained from Gene Expression Profiling Interactive Analysis (GEPIA) online database (http://gepia.cancer-pku.cn). CaM gene expression was high in LGGs and GBM compared with that in other tumor types. Moreover, the level of expression of *CALM1* and *CALM2* was elevated in GBM compared with that in LGGs (Fig. 2b). Data obtained from the human protein atlas showed a similar expression pattern, with prominent CaM genes expression in GBM compared with that in other tumor types (Supplementary Figure 3a–c).

Next, we analyzed the association between CaM expression and overall survival in the TCGA GBM data set. Kaplan–Meier analysis indicated that patients with high *CALM1* and *CALM2* expression in GBM tissues showed shorter overall survival than those with low *CALM1* and *CALM2* expression (*p* = 0.0011 and *p* = 0.0018; Fig. 2c). However, *CALM3* expression was not correlated with the survival of patients with GBM (*p* = 0.9531; Fig. 2c). Similarly, among 284 patients with GBM in the French GBM data set, 129 patients showed upregulation of CaM, confirming that high CaM expression is associated with poor prognosis. (*p* = 0.0059; Fig. 2d).

### CaM concentration is correlated with the invasive capacity and invadopodia activity of GBM cells

**Fig. 3:**
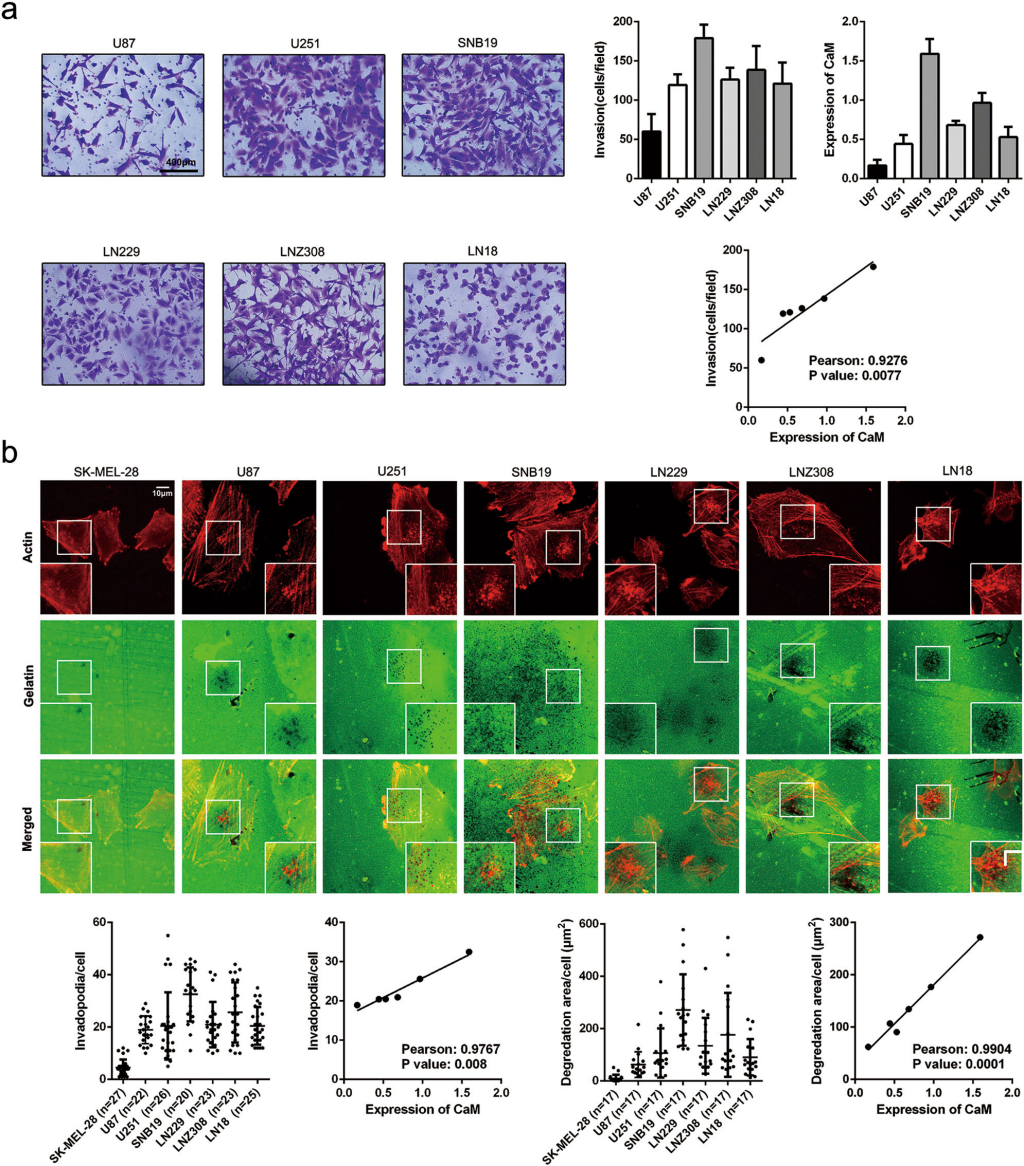
CaM content strongly correlates with invasion capacity and invadopodia formation in GBM cell lines. **a** Invasion of GBM cell lines were detected by transwell invasion assay. The correlation between CaM expression and invasion in GBM cell lines were determined using Pearson correlation analysis (the data are presented as means ± SDs of three different microscopic visions from every independent experiment). **b** The ability of invadopodia formation and gelatin degradation in GBM cell lines. Invadopodia formation and gelatin degradation strongly correlates with CaM expression in GBM cell lines (images were taken from three different microscopic visions from every independent experiment, Invadopodia formation: *n* = 27, 22, 26, 20, 23, 23 and 25 for SK-MEL-28, U87, U251, SNB19, LN229, LNZ308, LN18; Proteolysis activity: *n* = 17, 17, 17, 17, 17, 17 and 17 for SK-MEL-28, U87, U251, SNB19, LN229, LNZ308, LN18). Statistical analysis was performed by Pearson correlation analysis

To explore the correlation between CaM concentration and GBM invasion, we performed a transwell invasion assay and assessed the invasive capacity of GBM cell lines. Pearson correlation analysis was performed to evaluate the correlation between CaM concentration and GBM cell invasion. Results of Pearson correlation analysis showed a strong correlation between CaM expression and GBM cell invasion (Pearson correlation coefficient = 0.9276, *p* = 0.0077; Fig. 3a).

Invadopodia are suggested to be important for orchestrating tumor cell dissemination during metastasis^4^, and treatment with a CaM antagonist W7 effectively inhibits invadopodia formation^18^. Therefore, we hypothesized that CaM is critical for invadopodia formation. To confirm this, we performed an invadopodia activity assay and investigated invadopodia formation patterns of different GBM cell lines, with SK-MEL-28, a noninvasive melanoma cell line showing low gelatin degradation, as a negative control^19^. Invadopodia activity was evaluated by assessing focalized degradation areas in the different cell lines. Pearson correlation analysis showed that CaM expression was highly correlated with invadopodia formation (Pearson correlation coefficient = 0.9767, *p* = 0.008) and focalized degradation in GBM (Pearson correlation coefficient = 0.9904, *p* = 0.0001) (Fig. 3b). These results suggest that CaM orchestrates GBM invasion by facilitating invadopodia formation.

### CaM suppression impairs the invasion of and invadopodia formation by GBM cells

**Fig. 4:**
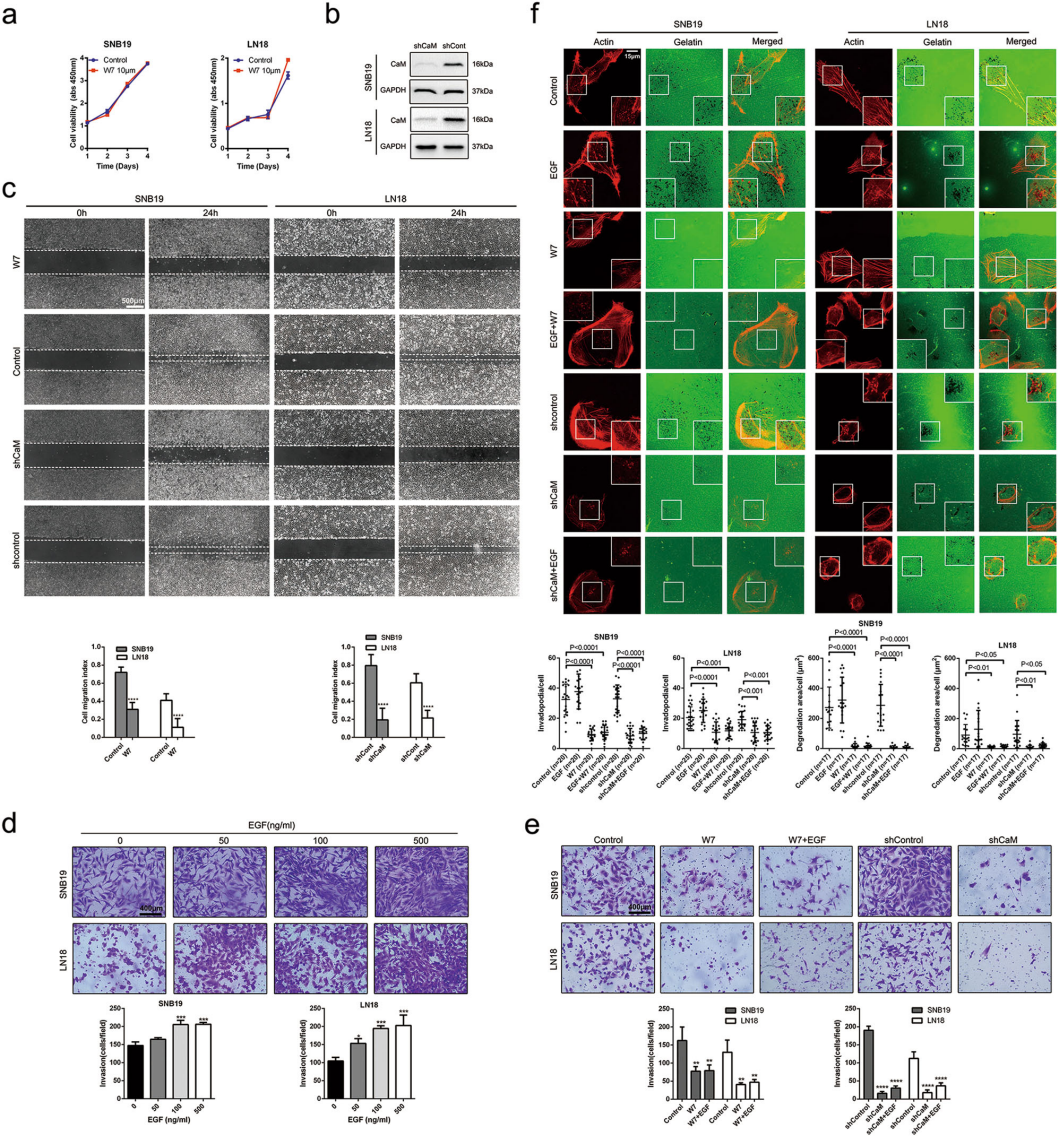
CaM inhibition impaired the invasion and invadopodia formation of SNB19 and LN18 cells. **a** The viability of SNB19 and LN18 cells treated by CaM-specific inhibitor W7 at 10μm was measured by cell counting kit-8 assay (Data are presented as means ± SDs of five different absorbance values from every independent experiment). **b** Expression of endogenous CaM in glioma cells was inhibited by expressing shRNAs targeting human *CALM1* and *CALM2* but not with the expression of nontargeting control shRNA (shCont). **c** Using W7 (10 μm) and CaM knockdown both significantly decreased GBM cells migration as detected by wound healing assay (Data are presented as means ± SDs of five different width of the wound from every independent experiment, ****p < 0.0001). **d** EGF facilitated GBM cells invasion in a dose-dependent manner analyzed by transwell invasion assay (Data are presented as means ± SDs of three different microscopic visions from every independent experiment, ****p < 0.0001). **e** Using W7 (10 μm) and CaM knockdown both significantly inhibited GBM cells invasion and EGF-induced invasion (**p < 0.01, ****p < 0.0001). **f** CaM inhibition abolished GBM cells invadopodia formation and focalized proteolysis activity and antagonized EGF-induced invadopodia formation and focalized proteolysis activity (Images were taken from three different microscopic visions from every independent experiment, Invadopodia formation: *n* = 20, 20, 20, 20, 20, 20 and 20 for Control, EGF, W7, EGF + W7, shcontrol, shCaM, shCaM + EGF in SNB19 and *n* = 25, 20, 20, 20, 20, 20 and 20 for Control, EGF, W7, EGF + W7, shcontrol, shCaM, shCaM + EGF in LN18; Proteolysis activity: *n* = 17, 17, 17, 17, 17, 17 and 17 for Control, EGF, W7, EGF + W7, shcontrol, shCaM, shCaM + EGF). Invadopodia quantification and degradation areas were measured by imagej (*p < 0.05, **p < 0.01, ***p < 0.001, ****p < 0.0001)

To confirm the involvement of CaM in GBM invasion and invadopodia formation, we inhibited CaM function using specific shRNAs or the CaM-specific inhibitor W7. We performed cell counting kit-8 assay to evaluate the viability of SNB19 and LN18 GBM cells treated with 10 μm W7, which has a moderate effect on downstream protein activity (Fig. 4a). To effectively suppress CaM expression in GBM, we obtained five sequences targeting the CaM genes and randomly selected two clones. A mixture containing equal amounts of these two clones was used to suppress CaM expression (Supplementary Figure 1). Finally, we selected the two most effective clones for silencing *CALM1* and *CALM2*, respectively (Fig. 4b).

Wound healing assay was performed to determine the effect of CaM inhibition after 10 μm W7 treatment on GBM invasion. CaM inhibition significantly inhibited the migration of SNB19 and LN18 cells, with the number of migrating cells in the CaM inhibition group being decreased by 40–60% compared with that in the control group (Fig. 4c).

Invadopodia formation is stimulated by various growth factors, with EGF being the best-characterized growth factor for stimulating invadopodia formation^4^. We performed transwell invasion assay to explore the role of EGF in GBM invasion. EGF promoted GBM cell invasion in a dose-dependent manner (Fig. 4d). We also performed the transwell invasion assay to investigate the effect of CaM inhibition on EGF-induced cell invasion. Results of the transwell invasion assays showed that CaM inhibition after 10 μm W7 treatment or CaM knockdown strikingly reversed and abrogated GBM cell invasion induced by 100 ng/ml EGF (Fig. 4e).

To determine whether CaM is involved in invadopodia formation, we performed an invadopodia assay by treating the cells with the CaM inhibitor W7 or shRNAs against CaMs. CaM inhibition after 10 μm-treatment of W7 or CaM knockdown effectively inhibited invadopodia formation and focalized degradation and significantly impaired invadopodia formation induced by 100 ng/ml EGF (Fig. 4f).

### Interactome of CaM with invadopodia-associated proteins

**Fig. 5:**
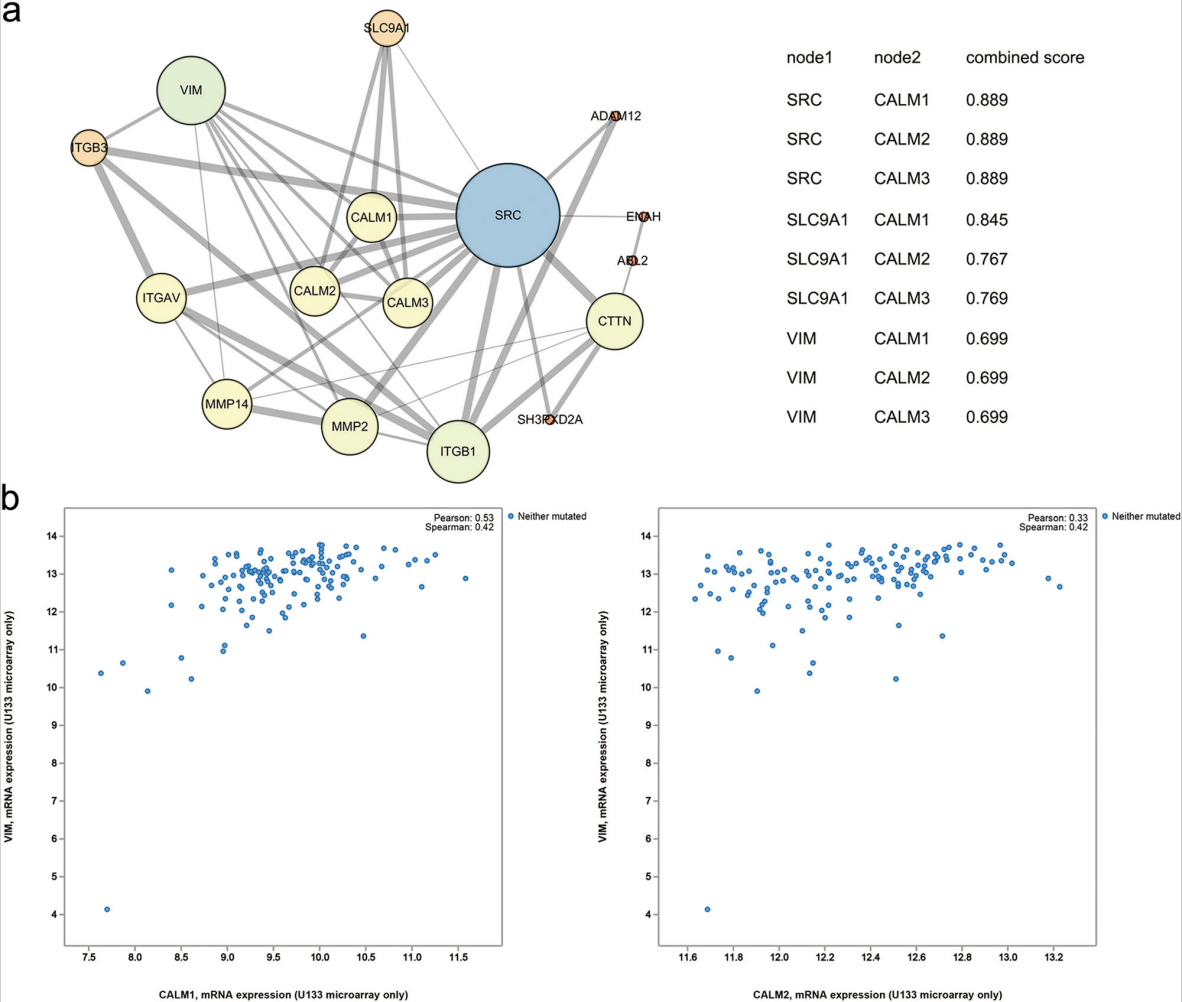
The interactome of CaM with invadopodium-associated proteins. **a** Known and predicted protein-protein interactions between CaM and invadopodium-associated proteins, the combined scores of direct associations between CaM and invadopodium-associated proteins were listed. The data were obtained from the STRING online database (https://string-db.org) and images were made using Cytoscape. **b** The coexpression of *CALM1* and *CALM2* with vimentin in 604 TCGA GBM samples. Data was from the cbioportal online database (http://www.cbioportal.org/)

The above results confirm that CaM plays a pivotal role in invadopodia formation. To investigate downstream regulatory mechanisms underlying the effect of CaM in invadopodia organization, we mapped an interactome of CaM with invadopodia-associated proteins using STRING (https://string-db.org) (Fig. 5a). Of the various invadopodia-associated proteins, Src, NHE1, and vimentin, which are suggested to be involved in CaM-induced invadopodia formation^3,4,20–22^, showed a clear, strong, and direct association with CaM.

A recent study suggested that epithelial–mesenchymal transition (EMT) is associated with pathways that initiate invadopodia formation^4^. Another study detected vimentin in mature invadopodia and reported that an intact vimentin network is required for invadopodia elongation^22^. Analysis of data derived from 604 TCGA GBM samples obtained from cibioportal (http://www.cbioportal.org/) indicated a positive correlation between vimentin and *CALM1* and *CALM2* (Fig. 5b).

### EGF potentiates CaM redistribution from the nucleus to the cytoplasm and its binding to Src and NHE1

Rapid CaM redistribution to subcellular compartments in response to various extracellular signals contributes to CaM-mediated regulation of different cellular processes^8,9^. Interaction of CaM with a microtubule motor induces its translocation to the leading edge of migrating cells concurrently with microtubule^3^. Elongation of invadopodia into basement membranes depends on a microtubule network that forms at the base of the structure, indicating that microtubules deliver components to the protruding tips of invadopodia^22^. Therefore, we postulated that CaM is delivered by microtubules to the base of invadopodia. Moreover, tumor cells exposed to an EGF gradient preferentially form invadopodia along the region facing the gradient^23^. Therefore, we examined whether EGF induced the spatial redistribution of CaM to the region of cells forming invadopodia.

**Fig. 6:**
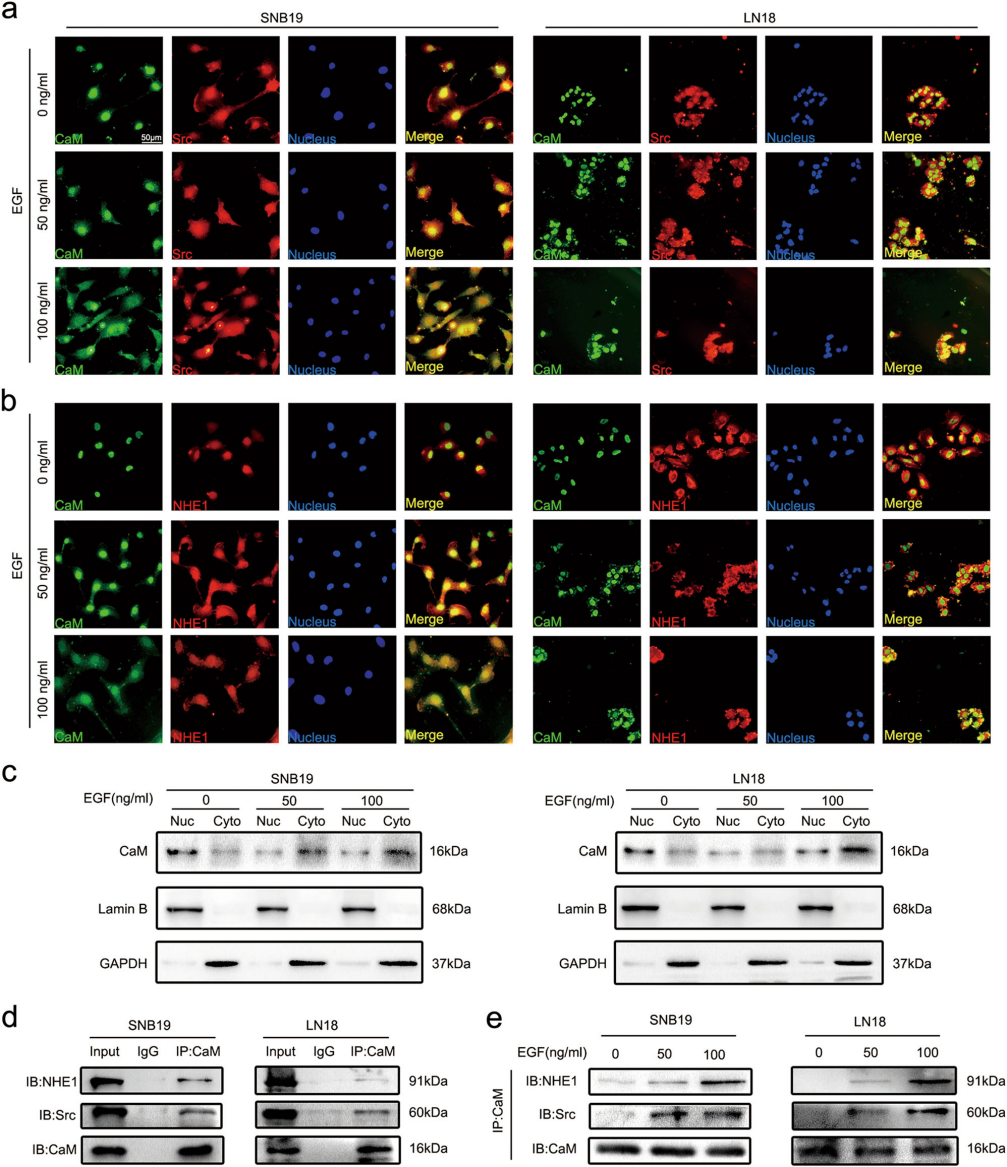
EGF potentiated CaM redistribution from nucleus to cytoplasm and binding with Src and NHE1. **a** EGF-induced CaM colocalization with Src in the cytoplasma by Immunofluorescence analysis. Green: CaM, Red: Src, Blue: Nucleus. (Representative images were presented. Images were taken from three different microscopic visions from every independent experiment). **b** EGF-induced CaM colocalization with NHE1 in the cytoplasma by Immunofluorescence analysis. Green: CaM, Red: Src, Blue: Nucleus. (Representative images were presented. Images were taken from three different microscopic visions from every independent experiment). **c** The distribution of CaM in nucleus and cytoplasm with different concentrations of EGF were detected by western blot. Lamin B1 and GAPDH served as nucleus (Nuc) and cytoplasmic (Cyto) loading control, respectively. **d** The combination between CaM and Src/NHE1 was detected by using co-immunoprecipitation assay without treatment with EGF. **e** Upon treatments with different concentrations of EGF, the binding of CaM with Src and NHE1 was explored by co-immunopreciptation

To explore whether EGF potentiated CaM translocation from the nucleus to the cytoplasm and its binding to invadopodia-associated proteins, we performed immunofluorescence analysis and determined the colocalization of CaM with Src and NHE1 in the presence of different EGF concentrations. Fluorescent signals of Src and NHE1 but not of CaM were clearly detected on the plasma membrane in the absence of EGF stimulation (Fig. 6a, b). After EGF stimulation, fluorescent signals of CaM clearly increased in the cytoplasm and those of Src and NHE1 remained unchanged (Fig. 6a, b).

To further confirm the effect of EGF on CaM translocation, we performed western blotting analysis to detect CaM concentration in the nucleus and cytoplasm in the presence of different EGF concentrations. EGF treatment induced CaM translocation (Fig. 6c), which was consistent with the results of immunofluorescence analysis.

Binding of CaM to Src and NHE1 is essential for their activation^24–27^. We performed a co-immunoprecipitation (co-IP) assay to investigate the interaction of CaM with Src and NHE1. CaM weakly interacted with Src and NHE1 in the absence of EGF (Fig. 6d) and strongly interacted with Src and NHE1 in a dose-dependent manner in the presence of EGF (Fig. 6e).

NHE1, an ion exchanger that increases intracellular pH by exchanging intracellular H^+^ with extracellular Na^+^ and an important factor for invadopodia assembly, is activated upon binding to CaM in different cancers^13,24,26,28^. Moreover, treatment with the CaM inhibitor W7 inhibits IL-6-mediated increase in intracellular pH^29^.

### CaM suppression inhibits Src and MMP activity and downregulates vimentin expression

**Fig. 7:**
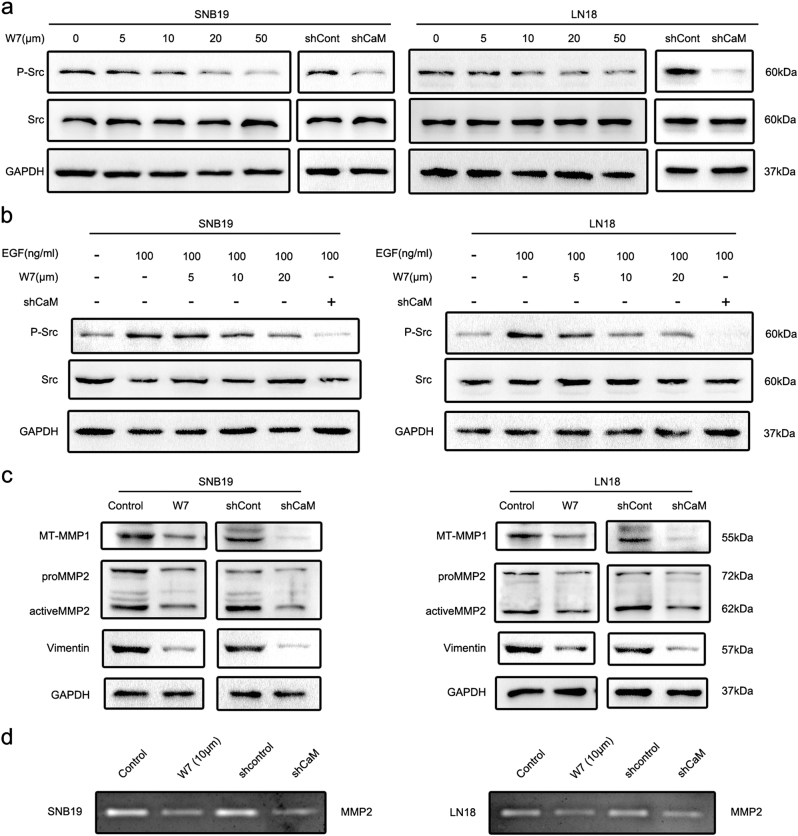
CaM suppression inhibited activity of Src and MMPs and downregulated vimentin expression. **a** Using W7 and CaM knockdown both significantly inhibited phosphorylation level of Src. **b** Using W7 and CaM knockdown abolished EGF-induced Src phosphorylation. **c** Using W7 (10 μm) and CaM knockdown both downregulated expression of vimentin and active form of MMPs. **d** Using W7 (10 μm) and CaM knockdown significantly inhibited MMP2 activity detected by Gelatin zymography assay

To understand molecular mechanisms underlying CaM-induced regulation of invadopodia formation, we investigated the effects of CaM on a panel of protein kinases and MMPs. To detect Src activity, we measured the levels of phosphotyrosine 416 Src. For this, GBM cells were treated with W7 or shRNAs for 3 h and inhibition of Src activity was determined (Fig. 7a). Next, we determined whether CaM inhibition impaired EGF-induced Src activation and found that both W7 treatment and CaM knockdown reversed EGF-induced Src activation (Fig. 7b).

Invadopodia contain three main classes of proteases, namely, zinc-regulated metalloproteases (MMP2, MMP9, MT1-MMP, and ADAM), cathepsin cysteine proteases, and serine proteases^3^. Several studies have shown that an active form of processed MT1-MMP produces a 55-kDa band^30^ and that an active form of MMP2 produces a 62 KDa band^31^. Therefore, we first analyzed the expression levels of active forms of MT1-MMP and MMP2 by performing western blotting analysis. W7 (10 μm) treatment or CaM knockdown decreased the levels of the active forms of MT1-MMP and MMP2 (Fig. 7c). To further verify our hypothesis, we performed a gelatin zymography assay to determine the effect of CaM suppression on MMP2 activity (Fig. 7d).

Vimentin, an EMT marker, is present in mature invadopodia and is required for invadopodia elongation^22^. In the present study, we found that W7-treatment or CaM knockdown decreased vimentin expression (Fig. 7c).

### CaM inhibition abolishes GBM invasion in vivo

**Fig. 8:**
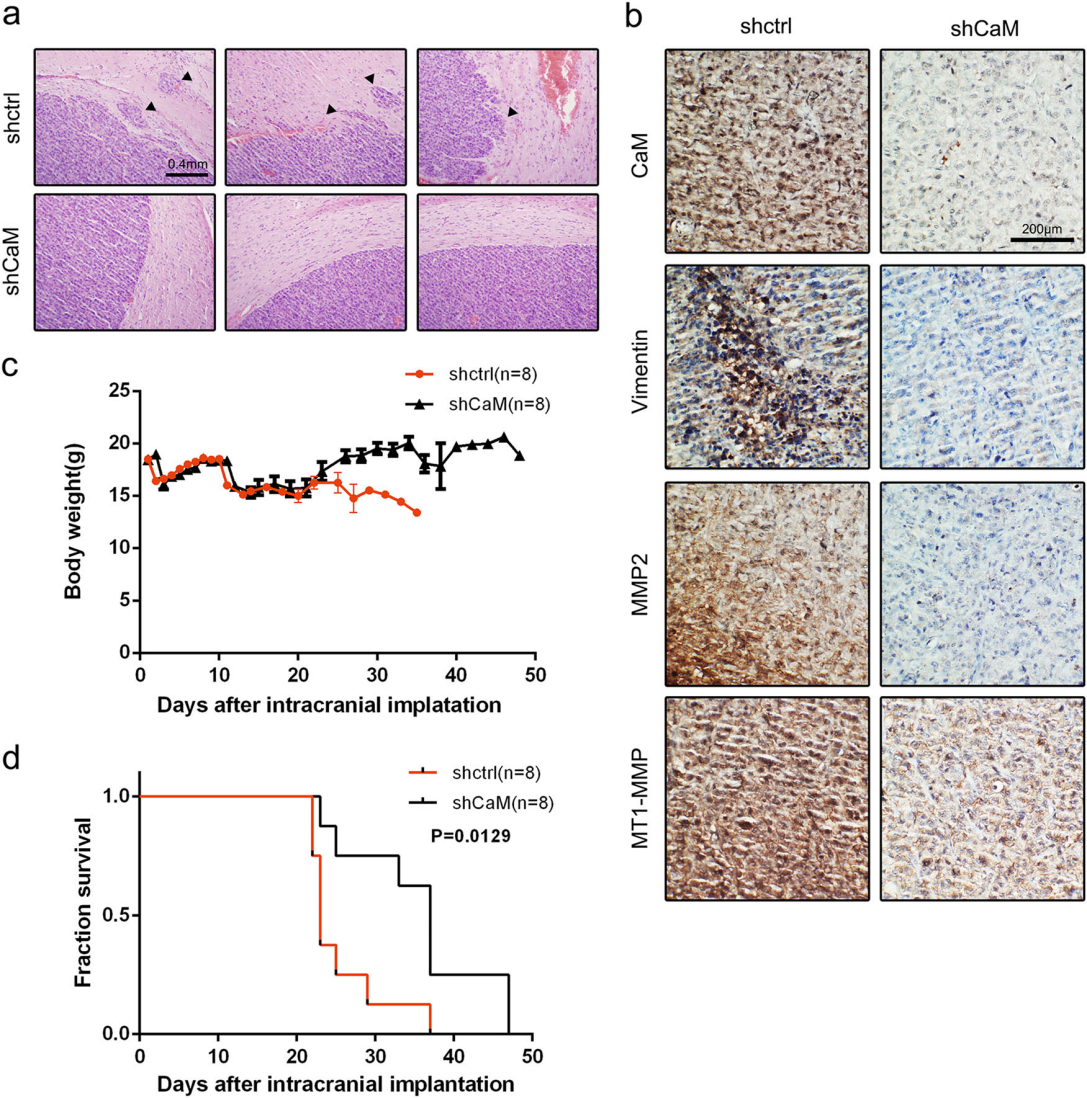
Silencing CaM impaired the invasion of GBM cells in vivo and prolonged the survival of GBM mice (CaM knockdown group: *n* = 8, control group: *n* = 8). **a** Representative images of H&E staining of tumor border from mice 3 weeks after tumor implantation (black triangle: disseminated tumor). **b** Immunohistochemistry analysis of the expression of CaM, MMP2, MT1-MMP and vimentin in CaM-shRNA-treated tumors compared to tumors in the control group (Representative images were presented. Images were taken from three different microscopic visions from every independent experiment). **c** Mouse weights were monitored in each group after tumor implantation. **d** Mouse survival in different groups was quantified by a Kaplan–Meier curve

Results of the in vitro experiments indicated that CaM inhibition impaired GBM cell invasion. To validate this result, we investigated the anti-invasive effect of CaM inhibition in vivo. We performed histological analysis of tumors isolated from xenografted BALB/cA nude mice divided in different groups at 4 weeks after tumor cell implantation by performing hematoxylin and eosin (H&E) staining and IHC analysis to detect tumor borders and downstream protein regulation. We observed that the tumors of control mice (*n* = 8) showed an invasive border while those of CaM-knockout mice (*n* = 8) showed a smooth border. In addition, we observed that invasive tumor cells formed island-like shapes with a colonizing morphology and aggregated together at the tumor border (indicated by a black triangle; Fig. 8a). Results of IHC analysis showed decreased CaM, MMP2, MT1-MMP, and vimentin expression, which was consistent with the results of in vitro experiments (Fig. 8b).

Body weights and survival duration of mice in the different groups were recorded after tumor cell implantation. At ~3 weeks after tumor cell implantation, control mice showed a sharp decrease in body weight and deterioration of health condition compared with CaM-knockout mice (Fig. 8c). Furthermore, CaM-knockout mice showed significantly longer survival than control mice (*p* = 0.0129; Fig. 8d).

## Discussion

In this study, we found that CaM redistribution promoted GBM invasion and ECM degradation by increasing invadopodia formation and activity. CaM inhibitor treatment and CaM knockdown effectively impaired tumor cell invasion both in vitro and in vivo, suggesting a potential approach for preventing GBM invasion.

Results of the histological analysis of tumor specimens showed elevated CaM protein expression in high-grade glioma specimens compared with that in normal tissue and LGG specimens. Moreover, CaM showed negative or weak expression in glial cells obtained from normal tissues and median or strong expression in glial cells obtained from glioma tissues, which was consistent with the data obtained from the human protein atlas. We also detected basal level of CaM expression in neuronal cells, indicating the regulatory role of CaM in neurotransmitter formation and metabolism in normal tissues^32,33^. Furthermore, CaM mRNA expression was upregulated in neurons compared with that in neural stem cells, which are precursors of GBM cells^34^. Results of western blotting and IHC analyses showed that total CaM protein concentration was significantly lower in normal tissue specimens than in glioma tissue specimens.

Analysis of the data obtained from the TCGA indicated that *CALM1* and *CALM2* were highly expressed in GBM and that their expression was significantly correlated with poor overall survival. To further confirm the clinical relevance of CaM, we performed IHC analysis of GBM specimens showing varying degrees of invasion. Analysis of MR images showed aberrant overexpression of CaM in GBM specimens showing tissue invasion. A recent study showed that the interaction of CaM with p68 RNA helicase promotes tumor cell migration^10^. Consistently, treatment with the CaM inhibitor W7 downregulates the expression of *MTS1* and *NM23*, which are metastasis-associated genes, in highly metastatic murine melanoma cells^35^. Bourguignon et al. showed that treatment with anti-CD44v_3_ antibody, cytochalasin D, and W7 but not with colchicine effectively inhibits invadopodia formation by breast cancer cells^18^. Together, findings of the abovementioned studies and the clinical results of the present study suggest that CaM promotes GBM invasion by promoting invadopodia formation.

In the in vitro experiments, we first profiled the infiltrative capacity of CaM in six GBM cell lines. Our results suggested a strong positive correlation between CaM expression and GBM cell invasion. Furthermore, results of the invadopodia assay showed that CaM concentration was significantly correlated with invadopodia formation and activity in the six GBM cell lines. Subsequently, CaM function was inhibited using the specific inhibitor W7 or through lentivirus-mediated knockdown of the CaM genes. We found that CaM inhibition after W7 treatment or CaM knockdown significantly inhibited GBM cell invasion and invadopodia formation. Human GBM cells lines, especially the highly tumorigenic but noninvasive cell line U87-MG, have been widely used for developing mouse xenograft models^36^. In the present study, we used a weakly tumorigenic but highly invasive GBM cell line LN18 to analyze molecular mechanisms underlying the role of CaM in tumor dissemination in vivo. Consistent with the results of our in vitro experiments, results of our in vivo experiments suggested that CaM inhibition strikingly impaired GBM invasion.

Among the various growth factors that induce invadopodia formation, EGF is the best-characterized growth factor that sufficiently stimulates invadopodia formation in the absence of serum^37,38^. Moreover, EGF receptor is mutated and shows amplified expression in glioblastoma cells. We found that EGF promoted the invasion of and potentiated invadopodia assembly in GBM cells. Furthermore, W7 treatment or CaM knockdown reversed EGF-induced GBM invasion and invadopodia formation.

CaM is a highly conserved ubiquitous protein that binds to and regulates several target proteins that perform different functions^39^. Analysis of CaM interactomes and several CaM complexes showed a strong correlation between CaM and invadopodia-associated proteins such as Src, NHE1, and vimentin. Among the invadopodia-associated proteins, Src has the most node connections, suggesting its prominent role in invadopodia regulation. Src kinase plays a pivotal role in invadopodia formation^3^, which is consistent with the results of STRING analysis. Moreover, Src activity is prominently regulated by CaM^25^. NHE1, an ion exchanger that increases intracellular pH by exchanging intracellular H^+^ for extracellular Na^+^ and an important factor for invadopodia assembly^4,20,21^, is activated upon binding to CaM^24,26^. Decreased extracellular pH promotes the delivery of soluble (MMP2 and MMP9) and insoluble (MT1-MMP) proteases to invadopodia^40^. Vimentin expression is associated with EMT^41^. Furthermore, vimentin is present in mature invadopodia, and an intact vimentin network is required for invadopodia elongation^22,42^. Primary results of the present study indicated that EGF-induced CaM translocation from the nucleus to the cytoplasm and its binding to Src and NHE1. Invadopodia are preferentially formed in the region of tumor cells exposed to an EGF gradient, and these invadopodia are required for chemotactic sensing^23^. This indicates an association between the pattern of CaM spatial redistribution, invadopodia initiation, and chemotaxis. The interaction of CaM with Src and NHE1 is essential for Src and NHE1 activation. We found that CaM suppression inhibited Src, downregulated vimentin expression, and reversed EGF-induced Src activation.

Invadopodia maturation involves the recruitment and activation of multiple pericellular proteases that promote ECM degradation^5,43^. During invadopodia maturation, invadopodia promote ECM degradation by coordinating the secretion of MMP2 and MMP9 and promoting the delivery and presentation of MT1-MMP to the tip of protruding invadopodia^3^. Our data suggest that W7 treatment or CaM knockdown decreases the levels of active forms of MMP2 and MT1-MMP. Moreover, results of gelatin zymography assay confirmed the inhibition of MMP activity.

**Fig. 9:**
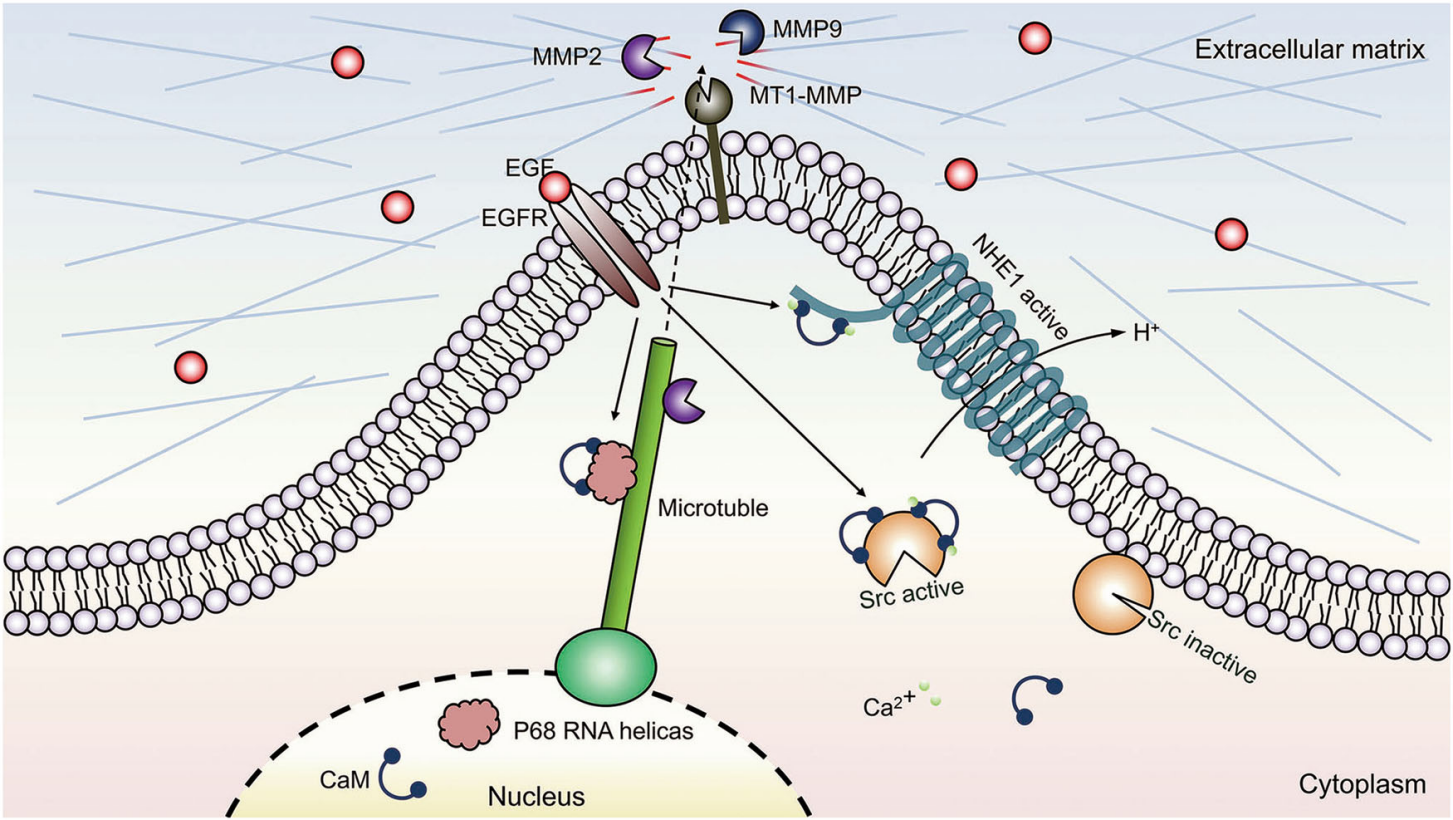
Model of CaM interactome in GBM invasion. A hypothetical model that illustrates the underlying mechanism of CaM in GBM invadopodia formation. After translocation to cytoplasm, CaM interacts with Src and NHE1 to potentiate the formation of invadopodia

Invasion is a critical step during GBM progression. The results of this study establish CaM as a central node in the molecular network that regulates invadopodia-associated proteins and MMP activity. Results of the present study also suggest that EGF-induced CaM redistribution from the nucleus to the cytoplasm facilitates the activation of invadopodia-associated proteins, leading to invadopodia formation and GBM invasion (Fig. 9), and that CaM is a candidate therapeutic target for impeding GBM invasion.

## Materials and methods

### Antibodies

Antibodies against CaM (ab2860; dilution for western blotting, 1:1000; dilution for IHC analysis, 1:50), NHE1 (ab67314; dilution for western blotting, 1:1000; dilution for immunofluorescence analysis, 1:100), MMP2 (ab37150; dilution for western blotting, 1:1000; dilution for IHC analysis, 1:100), and MT1-MMP (ab51074; dilution for IHC analysis, 1:100) were obtained from Abcam (UK). Antibodies against Src (36D10; dilution for western blotting, 1:1000; dilution for immunofluorescence analysis, 1:100), phosphorylated Src (Tyr416; dilution for western blotting, 1:1000), MT1-MMP (D1E4; dilution for western blotting, 1:1000), and vimentin (D21H3; dilution for western blotting, 1:1000; dilution for IHC analysis, 1:100) were obtained from Cell Signaling Technology (USA). Anti-lamin B1 antibody (12987-1-AP; dilution for western blotting, 1:1000) was obtained from Proteintech (USA), and anti-GADPH antibody (TA309157; dilution for western blotting, 1:2000) was obtained from ZSGB-Bio (China).

### Patients and GBM tissues

Freshly resected glioma tissues were collected from patients with WHO grade II (*n* = 9), grade III (*n* = 12), and grade IV GBM (*n* = 14) who were admitted to the Department of Neurosurgery, Tianjin Medical University General Hospital, China, from October 2011 to April 2017. Normal non-neoplastic brain tissues were obtained from patients with temporal lobe epilepsy (*n* = 6). Informed consent was acquired from all the patients. All experiments on human glioma tissues were approved by the Regional Scientific Ethical Committee.

### Cell lines

Human GBM cell lines U87-MG, U251-MG, LN229, LN18, SNB19, and LNZ308 and human melanoma cell line SK-MEL-28 were purchased from the Institute of Biochemistry and Cell Biology (Shanghai, China). The cells were cultured in Dulbecco’s modified Eagle medium (DMEM; Gibco, USA) containing 10% fetal bovine serum (FBS; Gibco, USA) at 37 °C in an incubator with an atmosphere of 5% CO_2_.

### Cell counting kit-8 assay

Viability of W7-treated cells was estimated by performing the cell counting kit-8 assay (DOJINDO, Japan), according to the manufacturer’s guidelines. SNB19 and LN18 cells (4000 cells per well) were seeded for 24 h in 96-well plates containing 100 μl medium in each well. Next, the cells were divided into different groups and were treated with 10 μm W7 for 24, 48, 72, and 96 h. Control cells were also divided into different groups and were cultured for 24, 48, 72, and 96 h. Next, the cells were incubated with 2-(2-methoxy-4-nitrophenyl)-3-(4-nitrophenyl)-5-(2,4-disulfophenyl)-2H-tetrazolium, mono-sodium salt (WST-8) for 2 h, and their absorbance was measured at 450 nm by using a microplate luminometer (BioTek, USA).

### H&E staining and IHC analysis

Paraffin-embedded tissues used for performing H&E staining and IHC analysis were prepared as described previously^44^. For performing IHC analysis, the paraffin-embedded tissues were cut into 6 μm-thick slices, fixed on a glass slide, deparaffinized in xylene, rehydrated in graded ethanol, treated with 0.3% hydrogen peroxide (ZSGB-Bio, China) for quenching endogenous peroxidase activity, and heated in an all-purpose powerful antigen retrieval solution (Beyotime, China) at 37 °C for antigen retrieval. Nonspecific proteins were blocked using 5% goat serum (Solarbio, China). Next, the slides were incubated overnight at 4 °C with the indicated primary antibodies. Immunostaining was determined using a two-step detection kit (ZSGB-Bio, China). Next, the slides were counterstained with Mayer hematoxylin solution (Solarbio, China) for nuclear staining. Images were obtained using VANOX microscope (Olympus, Japan) at ×200 magnification. Intensity score of immunostaining was graded as 0, negative; 1, weakly positive (light brown); 2, moderately positive (brown); and 3, strongly positive (dark brown), and quantity score of immunostaining was graded as 0, negative; 1, ≤25%; 2, 26–50%; 3, 51–75%; and 4, >75%. IHC score was calculated by adding the intensity and quantity scores.

For performing H&E staining, the paraffin-embedded tissues were cut into 6 μm-thick slices, fixed on a glass slide, deparaffinized in xylene, and rehydrated in graded ethanol. Next, the slides were stained with Mayer hematoxylin solution for nuclear staining, followed by staining with H&E by using an H&E staining kit (Solarbio, China). Images were obtained using VANOX microscope.

### Immunofluorescence analysis

Cells were seeded overnight on coverslips in a 12-well plate. After the cells reached 40% confluence, they were treated with different concentrations (0, 50, and 100 ng/ml) of EGF (Gibco, USA) for 3 h. Next, the cells were fixed with 4% paraformaldehyde for 10 min, permeabilized with 0.1% Triton X-100 for 10 min, blocked in 5% goat serum at room temperature for 30 min, and incubated overnight at 4˚C with the indicated primary antibodies. Next, the cell-seeded coverslips were rewarmed for 1 h and were incubated with specific secondary antibodies for 1 h at 37 °C. Nuclei were stained with DAPI for 5 min at room temperature. Finally, the coverslips were observed under a fluorescence microscope (Olympus, Japan).

### Western blotting analysis

Cells were lysed in RIPA buffer (Solarbio, China) containing PMSF (dilution, 1:100; Solarbio, China), and proteins were extracted from cells cultured in the absence of FBS. Before denaturation, total protein concentration was determined using BCA protein assay kit (Solarbio, China), according to the manufacturer’s instructions. The extracted proteins were separated by performing sodium dodecyl sulfate-polyacrylamide gel electrophoresis (SDS-PAGE), blotted onto PVDF membranes (Millipore, USA), and incubated overnight at 4˚C with the indicated primary antibodies. Next, the membranes were washed and incubated with goat anti-rabbit/mouse IgG secondary antibody (dilution, 1:2000) for 1 h at room temperature. Protein expression was analyzed using GBOX (Syngene Company, UK) and a chemiluminescent HRP substrate (Millipore, USA).

### Transwell invasion assay

Transwell invasion assay was performed using a Matrigel (BioCoat, USA) invasion apparatus (24-well format; BD Biosciences, USA). Briefly, 1 × 10^5^ cells were seeded in serum-free DMEM in the upper chamber of the apparatus, and the lower chamber was filled with DMEM containing 10% FBS as a chemoattractant. W7 or EGF was added to both the upper and lower chambers. After incubation for 24 h, non-invading cells in the upper chamber were removed using a sterile cotton swab. Transwell filters harboring the invading cells were fixed with 4% paraformaldehyde and stained with Giemsa solution. The number of invading cells present on the lower surface of the filters was counted.

### Wound healing assay

Glioma cells were cultured in a six-well culture plate until they reached 70% confluence. Next, the cell monolayers formed were scratched using a 200 μl sterile pipette tip. Cells in the W7-treated group were cultured in low-serum DMEM containing W7 and those in the control and CaM-knockdown groups were cultured in low-serum DMEM lacking W7. The cells were then allowed to migrate into the wounded area for 24 h, fixed with methanol, and stained with 5% Giemsa solution. Migrating index was calculated using the following formula: migration index = (width of the wound at 0 h—width of the wound at 24 h) × 100/width of the wound at 0 h^13^.

### Co-IP analysis

Co-IP analysis was performed as described previously^45^. Cultured cells was lysed, and their lysates were precleared using control IgG (Santa Cruz Biotechnology, USA) and protein A/G plus agarose (Thermo Fisher, USA). After centrifugation, supernatant obtained was used as a precleared protein lysate, treated with relevant primary antibodies, and incubated overnight at 4˚C with protein A/G plus agarose on a rotary shaker. Immunoprecipitates were collected by centrifugation and were gently washed with PBS. Bound proteins were resuspended in a loading buffer and were analyzed by performing western blotting analysis.

### Lentiviral shRNA transfection

For this, shRNAs against the CaM genes were generated using GV493 vector (hU6-MCS-CBh-gcGFP-IRES-puromycin; GeneChem, Shanghai, China). Sequences of the generated shRNAs are as follows: GAACCCAACAGAAGCTGAA (*CALM1*) and GCAGAGTTACAGGACATGA (*CALM2*). Cells were transfected with scrambled or shRNA-expressing lentiviral vectors, according to the manufacturer’s recommendations. After infection, stable cell clones transfected with the shRNA-expressing constructs were selected using 5 µg/ml puromycin solution. The cells were collected at 48 h after the transfection for performing further experiments.

### Invadopodia activity assay

FITC–gelatin-coated coverslips were prepared as described previously with a slight modification^46^. Briefly, a thin layer of FITC-conjugated gelatin was placed on the coverslips and was crosslinked with 0.8% glutaraldehyde on ice for 10 min. Crosslinking was continued at room temperature for additional 30 min. The coverslips were rinsed with PBS, incubated with 5 mg/ml sodium borohydride at room temperature for 3 min, rinsed again with PBS, incubated with 70% EtOH for 10 min, and dried at 37 °C for 15 min in a CO_2_ incubator. At 1 h before plating the cells, the coverslips were quenched with DMEM containing 10% FBS at 37 °C. The cells plated on the FITC–gelatin-coated coverslips were cultured in DMEM for 18 h to quantify invadopodia formation. Fluorescent micrographs were obtained using an upright fluorescence microscope (FV1200; Olympus, Japan) equipped with a 60× oil immersion objective. Invadopodia formation was determined by counting F-actin-rich dots of cells, and invadopodia activity was determined based on gelatin degradation in each cell using ImageJ software (National Institutes of Health, USA)^47^. A region of interest was selected along the outline of each cell based on phalloidin staining.

### Gelatin zymography assay

The proteolytic activity of MMP2 was determined using a gelatin zymography assay kit (Applygen, China), according to the manufacturer’s guidelines. Briefly, SNB19 and LN18 cells were cultured in a serum-deprived medium for 24 h. Next, 20 μl aliquot of the culture medium was resolved by performing SDS-PAGE on an 8% polyacrylamide gel containing 0.1 mg/ml gelatin. The gels were then incubated two times with 0.2% Triton X-100 for 30 min at room temperature (25 °C) to remove SDS and were rinsed five times with double-distilled water. Next, the gels were incubated with 20 mM NaCl, 5 mM CaCl_2_, 0.02% Brij-35, and 50 mM Tris/HCl buffer (pH 7.6) at 37 °C for 20 h; stained with 0.1% Coomassie brilliant blue R-250; and destained with 10% acetic acid and 30% methanol in water. Gelatinolytic activity was detected by identifying unstained bands on a blue background and was quantified by performing densitometric measurement.

### Nude mouse model carrying intracranial xenografts

In vivo experiments were performed using BALB/cA nude mice (age, 4 weeks) that were divided into CaM-knockout (*n* = 8) and control groups (*n* = 8). Mouse model harboring intracranial tumors was established by stereotactically implanting 0.5 × 10^5^ lenti-shNC- or lenti-shCaM-infected LN18 cells into the nude mice by using cranial guide screws (RWD Life Science, China). Body weights and overall survival of mice in both the groups were monitored every day. The brains of the mice were carefully extracted, fixed in 10% formalin, and embedded in paraffin for performing H&E staining to detect tumor borders. All animal experiments were approved by the Ethical Committee of the Tianjin Medical University General Hospital.

### Statistical analysis

All tthe data were examined at least three times. The quantitative data are expressed as mean ± s.d. All statistical analyses were performed using SPSS version 16.0 (IBM, USA), and a two-tailed *p* value of < 0.05 was considered statistically significant.

## Electronic supplementary material

Supplementary Table 1

Supplementary Figure 1

Supplementary Figure 2

Supplementary Figure 3

Supplementary Information
