# Supplementary material for: The interactome and spatial redistribution feature of Ca2+ receptor protein calmodulin reveals a novel role in invadopodia-mediated invasion
Source: Cell Death Dis. 2018 Feb 20;9(3):292. doi: 10.1038/s41419-017-0253-7 (PMC5833463; doi:10.1038/s41419-017-0253-7)
Supplement: Supplementary file 1 — Supplementary Table 1 [file 41419_2017_253_MOESM1_ESM.doc]

**Supplementary Table 1:** The clinical features of the glioma specimens included in this study

| **WHO grade** | II (n=9) | III (n=12) | IV (n=14) |
| --- | --- | --- | --- |
| Age (Year, Mean ± SD) | 40.11±13.72 | 47.25±13.42 | 55.36±10.27 |
| **Gender** |  |  |  |
| Male | 7 | 4 | 5 |
| Female | 2 | 8 | 9 |
| **Predominant side** |  |  |  |
| Left | 3 | 4 | 7 |
| Right | 6 | 8 | 7 |
| Middle | 0 | 0 | 0 |
| **Predominant location** |  |  |  |
| Frontal lobe | 5 | 3 | 3 |
| Temporal lobe | 4 | 8 | 9 |
| Parietal lobe | 0 | 0 | 2 |
| Occipital lobe | 0 | 0 | 0 |
| Pineal body | 0 | 0 | 0 |
| Saddle area | 0 | 0 | 0 |
| Thalamus | 0 | 0 | 0 |
| Cerebellum | 0 | 1 | 0 |
| Third ventricle | 0 | 0 | 0 |

Abbreviation: SD, Standard deviation

**Supplementary Table 2:** shRNA sequences used in this study

| **NO.** | **Length** | **Target Seq (5' to 3')** |
| --- | --- | --- |
| CALM1-shRNA | 19 | GAACCCAACAGAAGCTGAA |
| CALM2-shRNA | 19 | GCAGAGTTACAGGACATGA |
