# Supplementary figures and images for: The interactome and spatial redistribution feature of Ca2+ receptor protein calmodulin reveals a novel role in invadopodia-mediated invasion
Source: Cell Death Dis. 2018 Feb 20;9(3):292. doi: 10.1038/s41419-017-0253-7 (PMC5833463; doi:10.1038/s41419-017-0253-7)

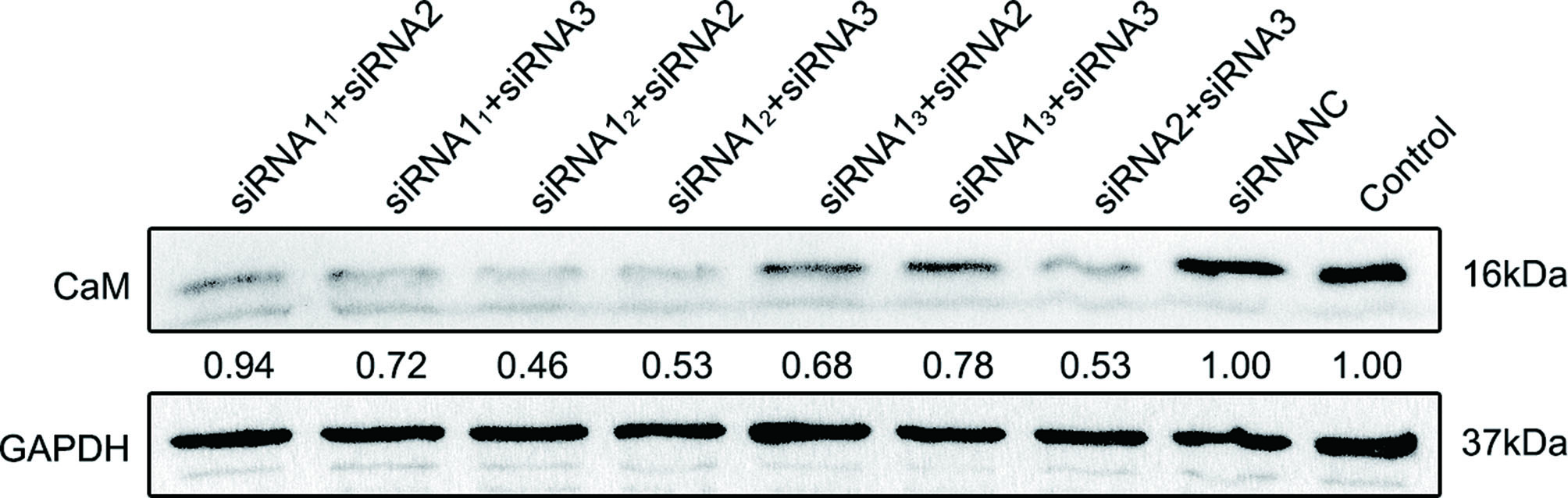

Supplement: Supplementary file 2 — Supplementary Figure 1 [file 41419_2017_253_MOESM2_ESM.jpg]

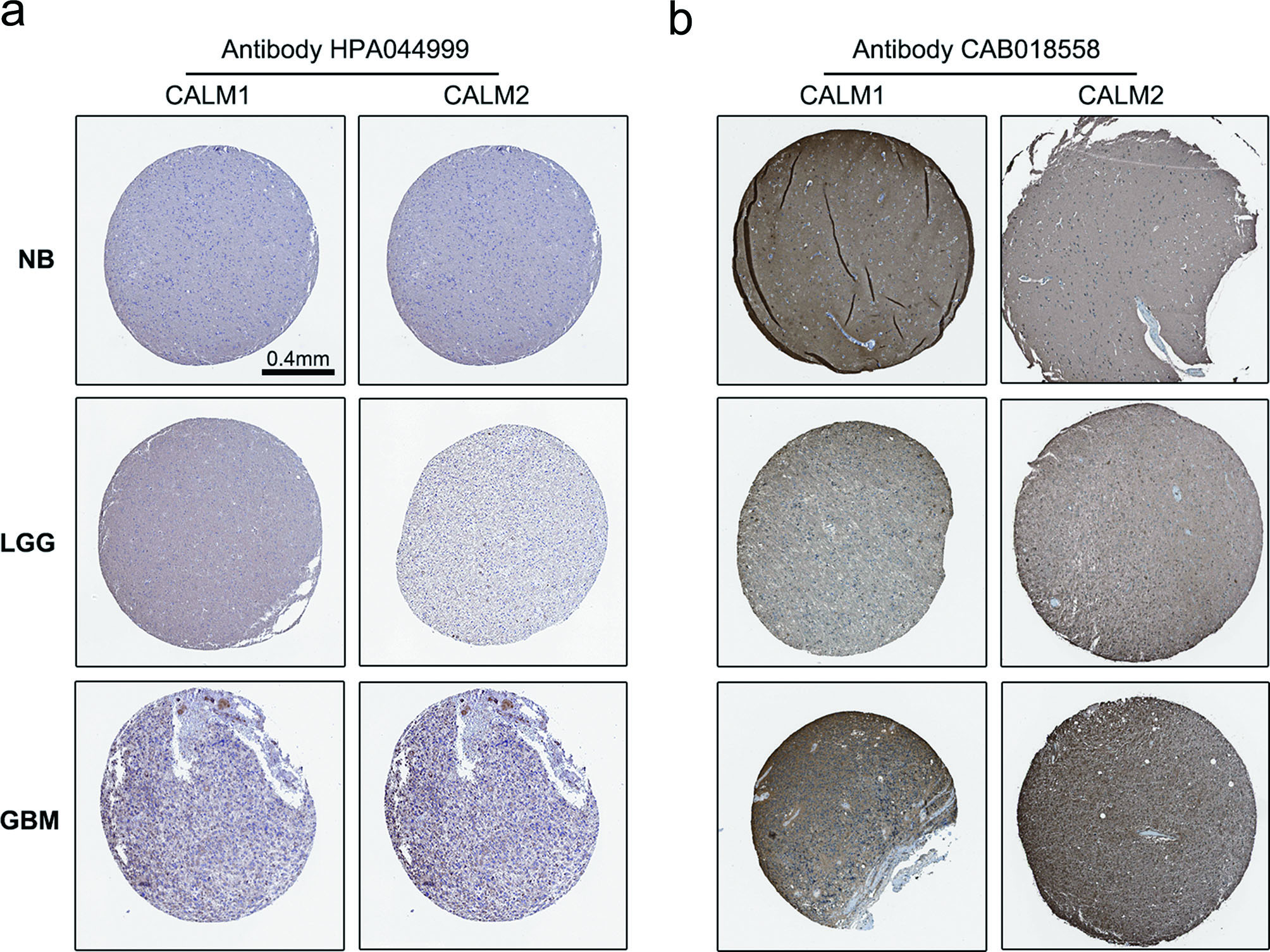

Supplement: Supplementary file 3 — Supplementary Figure 2 [file 41419_2017_253_MOESM3_ESM.jpg]

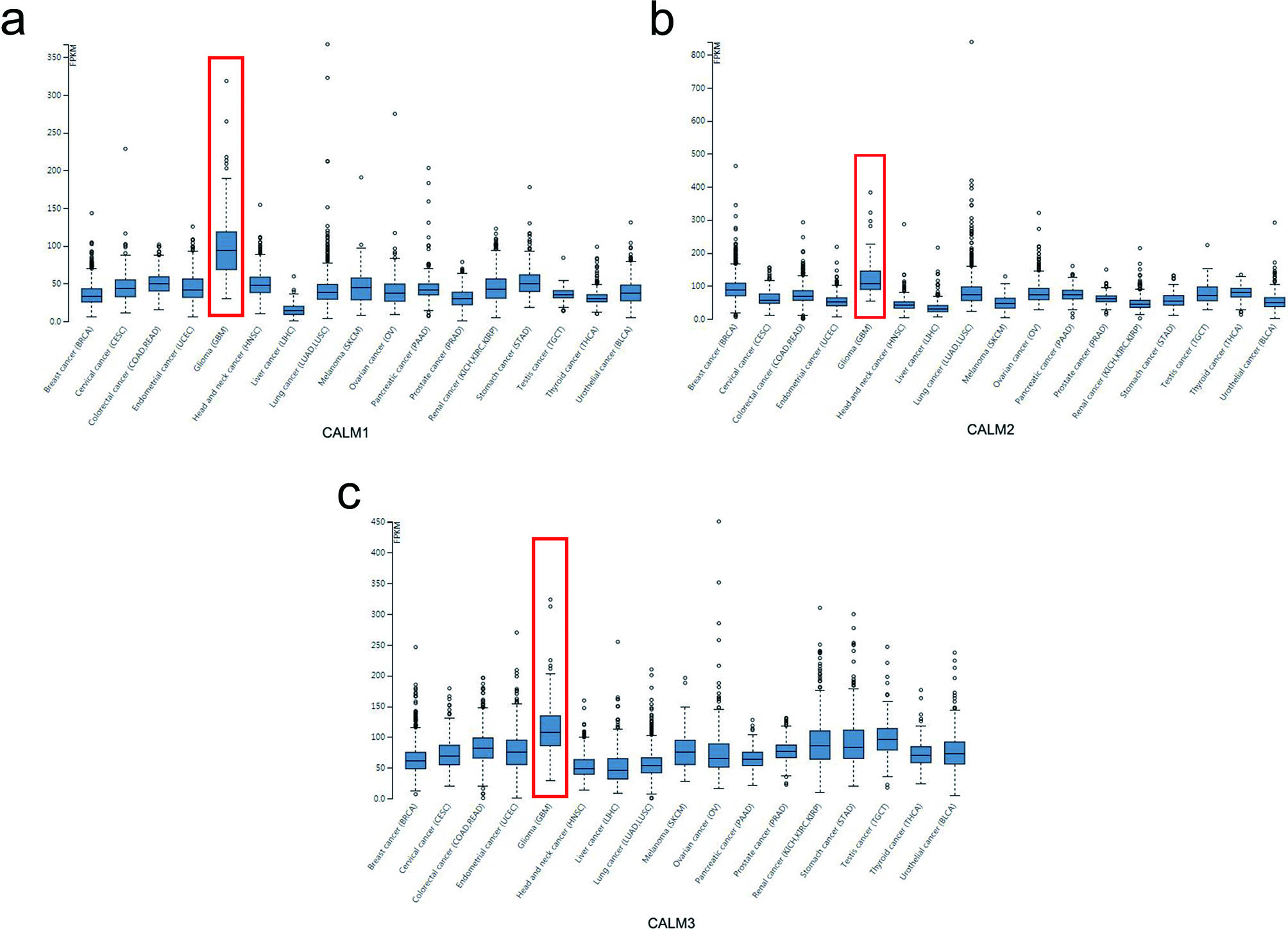

Supplement: Supplementary file 4 — Supplementary Figure 3 [file 41419_2017_253_MOESM4_ESM.jpg]
