# Supplementary material for: The interactome and spatial redistribution feature of Ca2+ receptor protein calmodulin reveals a novel role in invadopodia-mediated invasion
Source: Cell Death Dis. 2018 Feb 20;9(3):292. doi: 10.1038/s41419-017-0253-7 (PMC5833463; doi:10.1038/s41419-017-0253-7)
Supplement: Supplementary file 5 — Supplementary Information [file 41419_2017_253_MOESM5_ESM.docx]

**Supplementary Figure Legends**

**Supplementary Figure 1** Western Blot detected CaM expression in SNB19 cells after CaM knockdown treatment. To effectively suppress the expression of CaM in GBM, five sequences target to CaM genes were obtained and randomly selected two clones. A mixture of equal amounts of these two most effective clones was included in this study.

**Supplementary Figure 2** The expression of CaM in glioma samples. CALM1 and CALM2 expression in clinical specimens detected by two different antibodies (HPA044999, CAB018558) from the human protein atlas (www.proteinatlas.org). A negative or weak expression of glial cells in normal tissues and LGG, whilst a positive median or strong expression in GBM cells was shown.

**Supplementary Figure 3** CaM genes was prominently expressed in GBM. (a) From the data from human protein atlas (www.proteinatlas.org), CALM1 was prominently expressed in GBM compared with other tumor types. (b) From the data from human protein atlas (www.proteinatlas.org), CALM2 was prominently expressed in GBM compared with other tumor types. (c) From the data from human protein atlas (www.proteinatlas.org), CALM3 was prominently expressed in GBM compared with other tumor types.
